# Pathogens and Pathogenesis in Wheezing Diseases in Children Under 6

**DOI:** 10.3389/fonc.2022.922214

**Published:** 2022-07-14

**Authors:** Yongjun Tang, Yaxiong Yang, Ruohui He, Rong Huang, Xiangrong Zheng, Chentao Liu

**Affiliations:** ^1^ Department of Pediatrics, Xiangya Hospital, Central South University, Changsha, China; ^2^ Department of Pharmacy, Ningyuan County of People’s Hospital, Yongzhou, China

**Keywords:** wheezing, infant, IgE, IL-4, IFN-γ, MMP, IL-17

## Abstract

Few studies have comprehensively assessed the roles of cytokine production in wheezing pathogenesis. Therefore, we undertook this study to determine the association between wheezing episodes and cytokines, and to provide further information on this topic. Firstly, we retrospectively collected I176 children, including 122 subjects with first wheezing and 54 subjects with recurrent wheezing, to analyze the etiology and clinical characteristics of children with wheezing diseases. Then, we collected 52 children with wheezing diseases and 25 normal controls to detect the expression of interferon-γ (IFN-γ), interleukin-4 (IL-4), IFN-γ/IL-4, IL-17A, IL-17E, IgE, matrix metalloproteinase-3 (MMP-3), and MMP-9 in serum or plasma. The results showed that boys under 3 years old with history of allergies were more likely to develop wheezing diseases. In our cohort, M. pneumoniae caused a greater proportion of wheezing in children than expected. The expression of IgE [18.80 (13.65-31.00) vs. 17.9 (10.15-21.60)], IL-4 [24.00 (24.00-48.00) vs. 23.00 (9.50-27.00)], IFN-γ [70.59 (41.63-116.46) vs. 49.83 (29.58-81.74)], MMP3 [53.40 (20.02-128.2) vs. 30.90 (13.80-50.95)], MMP9 [148.10 (99.30-276.10) vs. 122.10 (82.20-162.35)], IL-17A [80.55 (54.46-113.08) vs. 61.11 (29.43-93.87)], and IL-17E [1.75 (0.66-2.77) vs. 1.19 (0.488-2.1615)] were significantly increased in the wheezing group (*p*<0.05) compared to normal controls, while the level of IFN-γ/IL-4 had no significant difference between the two groups (1.24 ± 1.88 vs 0.68 ± 0.74, *p*>0.05). There was altered cytokine production in children with wheezing diseases which was quite similar to asthma pathogenesis. Sex, age, pathogen infection, and inflammation in our study were also risk factors for wheezing diseases.

## Introduction

Wheezing respiratory illness is one of the most common diseases of childhood, with waves of prevalence in winter and summer. Wheezing infants always presented clinical manifestations such as a cough, fever, or shadows in chest X-rays. Approximately one-third of children under 3 years of age had had wheezing experiences, and over half of the participants who had been hospitalized for wheezing episodes had asthma in young adulthood (1, 2). Recurrent wheezing episodes greatly influence the maturation of children’s immune and respiratory systems, increase the risk of developing asthma in the future, and place a heavy burden on families and society.

There are many causes of wheezing diseases, such as infections, allergies, congenital anatomical deformities, and environmental and genetic factors. Infections can be caused by viruses, mycoplasmas (MP), bacteria, fungi, and other pathogens alone or in combination. Wheezing caused by different etiologies show different disease rules, and corresponding treatment plans and prognoses are also different. Martinez et al. (3) enrolled 725 children and found that patients with an allergic disease were more likely to develop another kind of allergic disease. Van’s follow-up study of a total of 6491 patients with allergic rhinitis underwent in 8.4 years showed that allergic rhinitis was an independent risk factor for asthma as patients with allergic rhinitis were five times more likely to develop asthma than people without (4). Ronmark and his colleagues carried out a survey with a questionnaire with 30,000 subjects in Switzerland that suggested that the co-existence of asthma, rhinitis, and eczema was common. Allergies, a history of asthma in the family, and smoking were all risk factors for eczema and the patients were characterized by wheezing. Therefore, finding out the risk factors of children with wheezing diseases is very important.

Several studies have underlined the association between pathogen infections and wheezing episodes, as children who had a virus, MP, bacteria, or fungal infection are more vulnerable to developing wheezing diseases (5). The altered cytokine production was also reported to be involved in the pathogenesis of MP-associated asthma. Hahn and his colleagues (6) identified that one-third of newborn infants with MP infection will develop asthma and the MP-specific IgE was detected in blood, nasopharyngeal, and bronchial secretion in half of the children with asthma. Another study undertaken on mice showed that bronchial hyperresponsiveness was alleviated on the third day of MP infection. On the fifth day, the ratio of interferon-γ(IFN-γ)/interleukin-4(IL-4) increased, but it decreased during 9-16 days indicating that the helper T lymphocyte 1 (Th1) cells played a major role at the initial stage of infection and Th2 cells at the later stage (7). The imbalance of CD4 helper T cell (Th) function was thought to be the most important mechanism, in which Th1 specific IFN-γ was decreased while the Th2 specific cytokine, IL-4, was observed to be increased. IL-4 functioned to enhance the proliferation of B lymphocytes and activate the synthesis of lgE in serum which was the mediator of rapid-type allergy reaction. Besides, the level of other cytokines like matrix metalloproteinase-3 (MMP-3), matrix metalloproteinase-9 (MMP-9), and interleukin- 17 (IL-17) were reported to participate in Th1/Th2 imbalance. However, whether they were involved in the pathogenesis of wheezing diseases was still unknown (8, 9).

To understand the differences and provide suggestions for the treatment of wheezing in children with different phenotypes, we conducted a retrospective study on 176 infants with wheezing diseases enrolled from Pediatrics department of Xiangya Hospital. In addition, we collected 52 subjects and 25 control subjects who displayed no wheezing from Xiangya Hospital and Hunan Children’s Hospital and aimed to reveal the association between cytokines (including lgE, IL-4, IFN-γ, IL-4/IFN-γ, MMP3, MMP9, IL-17A, and IL-17E) and wheezing illness.

## Materials and Methods

### Study Population

#### Cases From Xiangya Hospital

**Figure 1:**
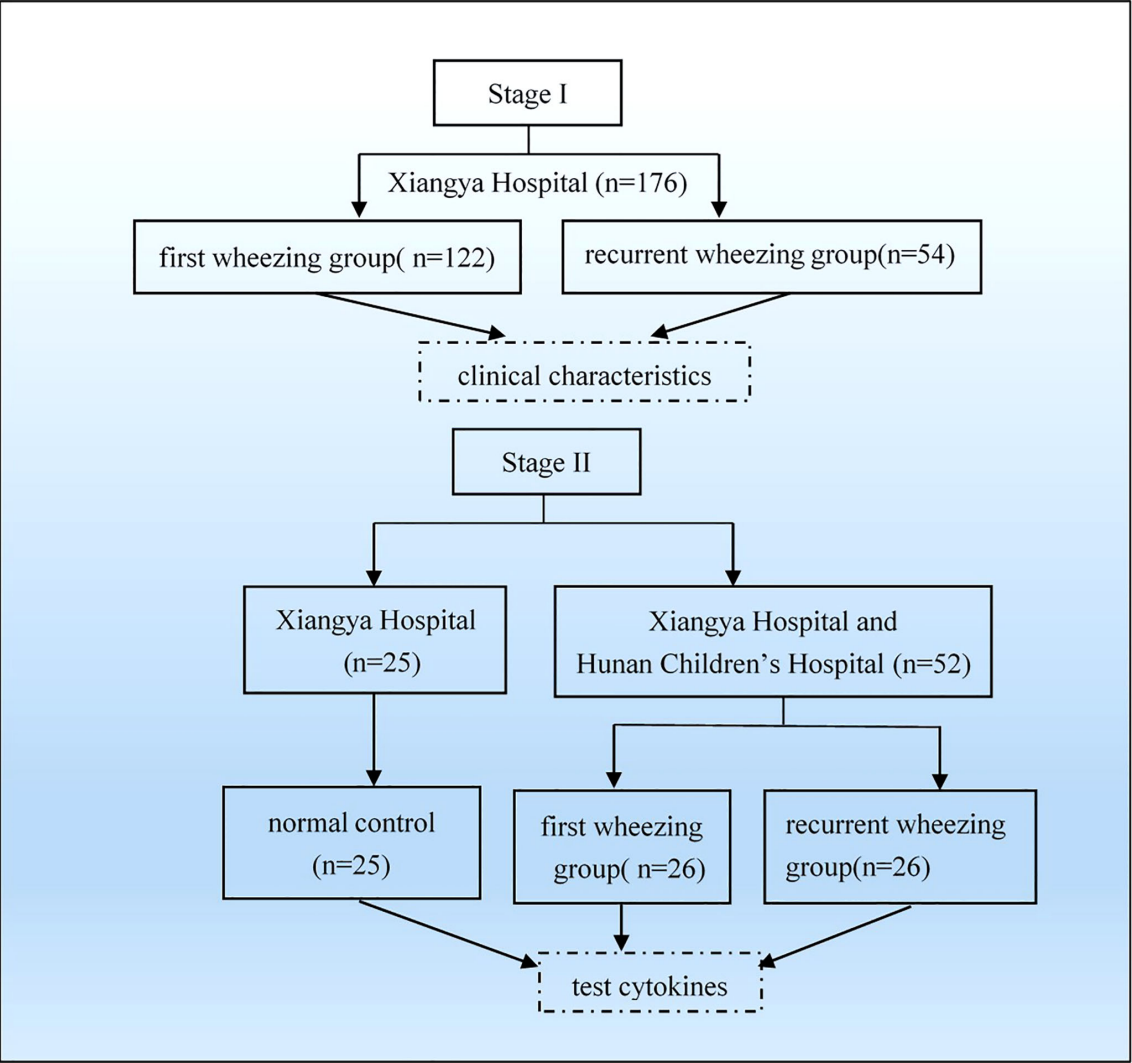
Flowchart of grouping and analysis. Firstly, we collected 176 patients from Xiangya Hospital and grouped as first wheezing (n = 122) and recurrent wheezing (n = 54) to analyze clinical characteristics. Then we collected 52 patients from Xiangya Hospital and Hunan Children’s Hospital and 25 normal control from Xiangya Hospital to test cytokines.

We recruited 176 children with wheezing diseases between September 2013 and April 2014 in the department of pediatrics, Xiangya Hospital. To be included in the study, had to have been hospitalized and diagnosed with asthmatic bronchitis and asthmatic pneumonia (10). Subjects were excluded from the study if: ① they had been born prematurely or born with respiratory malformation and heart disease; ② they had a primary immunodeficiency disease; ③ they had used hormones or immunosuppressive drugs less than 4 weeks before admission. The patients were aged from 1 month to 6 years (**Figure 1**).

Fifty-two subjects with wheezing illness were collected between December 2014 and February 2015. The inclusion and exclusion criteria were similar to the above. Twenty-five children who displayed no wheezing symptoms were enrolled as a control in this study. The control was defined as infants who had no history of wheezing, allergies, recurrent respiratory infections, use of glucocorticoids and immunosuppressive agents, other severe diseases, or disability.

### Data

The questionnaire was designed to collect clinical data including name, sex, age, telephone number, admission time, clinical symptoms, allergy history of the individual and their family, auxiliary measure of inspection, chest X-rays, and the treatment programs. A chest CT was conducted on specific patients.

### Viral Diagnostics

Blood samples were analyzed for respiratory viruses including coxsackie virus, respiratory syncytial virus (RSV), cytomegalovirus (CMV), Epstein-Barr virus (EB virus), rubella virus, and adenovirus by using standard techniques. The presence of lgM antibodies was considered indicative of viral infection.

### Bacterial Diagnostics

Bacterial antibodies were performed on serum, bone marrow, and sputum samples. Details of bacterial culture were reported previously and the species were identified according to standard methods (11).

### MP Diagnostics

Specimens were obtained from subjects using throat swabs and serum. *M. pneumoniae* was measured as previously reported and the patients were diagnosed with MP infection when anti-mycoplasma antibody titers were ≥1: 80 (12).

### Cytokine Assays

IL-4, IFN-γ, and IL-17 were detected on frozen serum samples using Human Cytokine/Chemokine Magnetic Bead Panel (Millipore, USA, Billerica, Massachusetts). The well was pre-wet in 200 μL Assay Buffer, then mixed on a plate shaker for 10 min. We removed the wash buffer thoroughly and added 25 μL of control or standard to the wells regarding Assay buffer as 0 pg/ml standard. 25 μL was added to the wells of samples. Next, we add 25μL serum matrix to the background, standards, and control wells and 25 μL serum sample to sample wells. We vortexed the mixing bottle and added 25 μL of the mixed beads to each well. Hereafter, we sealed and wrapped the plates and incubated them at 4°C for 16-18 h. We removed the well content and washed the plate with 200 μL Assay buffer per time and added 25 μL detection antibodies afterward. The plates were incubated at room temperature for 1 h and 25 μL Streptavidin-Phycoerythrin was added per well and then incubated at room temperature. The well content was removed and washed with 200 μL wash buffer two times then150 μL sheath fluid or drive fluid was added per well, and the concentrations of cytokine were detected on Luminex^®^ 200™, HTS, FLEXMAP 3D^®^, or MAGPIX^®^ with xPONENT^®^ software.

### MMP3 and MMP9 Measurements

The concentrations of MMP3 and MMP9 in serum or plasma samples were measured using enzyme immunoassay kits (R&D Systems, USA). Assays were performed following the manufacturer’s protocol. The data were analyzed using the standard curve-fitting method for calculating MMP3 and MMP9 concentrations in samples.

### Data Analysis

SPSS 26.0 statistical software was used for data analysis. Data ae shown as mean ± SD. χ^2^ test for proportions was used to assess the comparability between different sex and age groups. The data of lgE, IL-4, IFN-γ, MMP3, MMP9, IL-17A, and IL-17E were analyzed using the Rank sum test. The comparability of IL-4/IFN-γ between control and wheezing patients used an analysis of variance. Values of p<0.05 are considered significant.

## Results

### Characteristics of Children With Wheezing

A total of 176 children were enrolled in Xiangya Hospital cases, including 129 males and 47 females. The ratios of male to female in the first wheezing group and the recurrent group were 2.6:1 and 3.2:1, respectively. Boys in the first wheezing group and the recurrent wheezing group had more morbidity than girls, but there was no significant difference in gender between the two groups (*p* > 0.05, **Table 1**).

**Table 1:** Clinical data of 176 wheezing children from Xiangya Hospital.

	Total	First wheezing	Recurrent wheezing	*P*-value
Subjects (M/F)	176 (129/47)	122 (88/34)	54 (41/13)	0.600
Positive history of allergy	51	26 (21.3%)	25 (46.3%)	0.001
Positive family history of asthma	25	17 (13.9%)	8 (14.8%)	0.877
MP infection	38 (21.6%)	30 (24.6%)	8 (14.8%)	0.146

M, male; F, female; P-value was based on χ^2^ test.

The cases from Xiangya Hospital and Hunan Children’s Hospital included 26 first wheezing patients (19 males and seven females) with an average age of 11.35 ± 8.91 months, recurrent wheezing children (21 males and five females) with an average age of 13.96 ± 9.67 months. As for the normal control group, there were 19 male children and six female children with an average age of 13.92 ± 9.67 months. There is no significant difference in gender and age ratio in cases from Xiangya Hospital and cases from Xiangya Hospital and Hunan Children’s Hospital (*p* > 0.05, **Table 2**).

**Table 2:** Characteristics of cases and control from Xiangya Hospital and Hunan Children’s Hospital.

	No-wheezing control	Wheezing infants			
		Total	First wheezing	Recurrent wheezing	*p*-values
Subjects (M/F)	25 (19/6)	52 (40/12)	26 (19/7)	26 (21/5)	ns**^a^**
Age, months	13.92 ± 9.67	12.98 ± 9.29	11.35 ± 8.91	13.96 ± 9.67	ns**^a^**

**^a^** χ2 test for comparison of first wheezing group versus recurrent wheezing group.

### Association Between Allergy History and Wheezing

Fifty-one infants in cases from Xiangya Hospital had allergy history (mainly referred to as eczema). Among them, one was diagnosed with allergic rhinitis, one had urticaria, one was allergic to milk, and one was allergic to medicine (Penicillin G). Besides, in the first wheezing group, 26 children were allergy history-positive, while in the recurrent wheezing group, 25 children were allergy history-positive. Incidence of wheezing illness was strongly related to a history of allergy (21.3% vs. 43.6%, *p*=0.001) (**Table 1**).

### Wheezing and Family History of Asthma

By analyzing the cases in Xiangya Hospital, we found that 17 (13.9%) and eight (14.8%) children were asthma family history-positive in the first wheezing group and recurrent wheezing group, respectively, which indicated that there was no significant association between wheezing and family history of asthma. (**Table 1**).

### Wheezing and Pathogen

**Figure 2:**
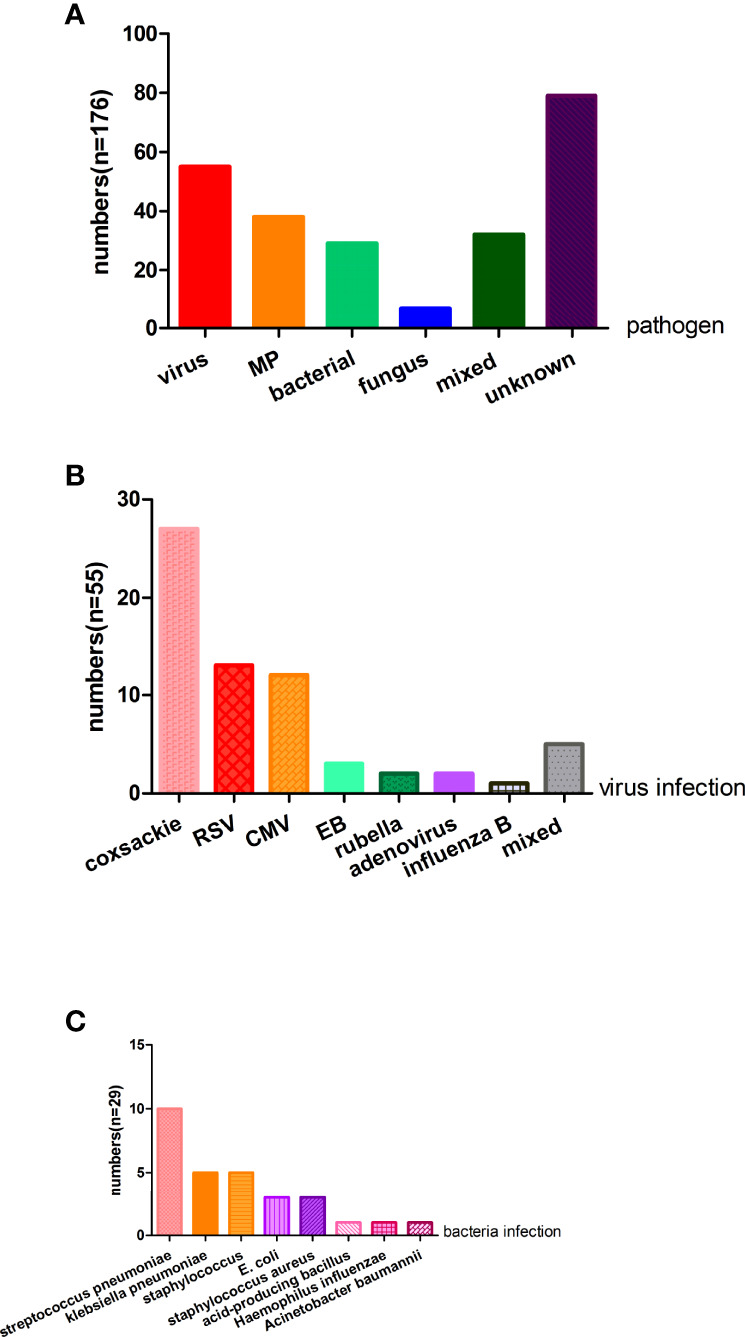
The pathogen analysis of the wheezing children **(A)** For 176 patients, virus, MP, bacterial, fungus, and mixed are causes of wheezing, but nearly half of the children’s etiology were unknown. **(B)** Fifty-five patients were infected with a virus and the top three virus were coxsackie virus, RSV, and CMV. **(C)** Twenty-nine patients were infected with bacteria and common bacteria were streptococcus pneumoniae, klebsiella pneumoniae, staphylococcus, and E.coli. MP, mycoplasma pneumoniae; RSV, respiratory syncytial virus; CMV, cytomegalovirus; E.coli, Escherichia coli.

Of the samples collected in Xiangya Hospital, 97 (55.11% of all the individuals) children were infected with a virus, MP, bacteria, or fungus. Fifty-five (31.3%) patients were infected with a virus, 38 (21.6%) were infected with MP, 29 (16.5%) patients were infected with bacteria, and 32 (18.2%) patients had more than one infection, respectively. Additionally, only seven (4.0%) patients in our study were infected with fungus. Twenty-seven (15.3%) infants were infected with coxsackie virus, 13 (7.4%) subjects were infected with RSV, 12 (6.8%) individuals were infected with cytomegalovirus, three (1.7%) patients were infected with EB virus, two (1.1%) patients were infected with rubella virus and two with adenovirus (1.1%), respectively. Only one (0.6%) individual was infected with influenza B virus. Moreover, three (1.7%) patients were infected with both the coxsackie virus and cytomegalovirus, one (0.6%) infant was infected with RSV and cytomegalovirus, and one infant was infected with RSV and EB virus (**Figure 2**).

Thirty subjects in the first wheezing group were infected with MP, along with eight patients in the recurrent wheezing group who had MP infection. However, no significant difference (24.6% vs. 14.8%, *p*>0.05) was found between the two groups (**Table 1**). Of the patients aged from 1 month to 1 year, 22 of the samples were infected with MP, 12 of the subjects who were 1 year old to 3 years old had MP infection, and four of the samples aged from 3 years old to 6 years old were infected with MP (**Table 3**). There was no significant difference among three age groups (20.0% vs. 25.0% vs. 22.2%, *p*>0.05). There were 17 children with MP infection in the allergy history-positive group and 21 children with MP infection in the allergy history-negative group. There was a significant difference (33.3% vs. 16.8%, *p*<0.05) between the two groups (**Table 4**).

**Table 3:** Age distribution of all and MP-infected subjects from Xiangya Hospital.

Age groups	All subjects	MP-infected subjects
1month to 1 year old	110 (62.5%)	22 (20.0%)
1~3 years old	48 (27.3%)	12 (25.0%)
3~6 years old	18 (10.2%)	4 (22.2%)
Total	176 (100%)	38 (21.6%)

χ2 = 0.4937, p>0.05.

**Table 4:** The comparison of the ratio of MP infection between groups with and without a history of allergy.

Groups	Number of subjects with MP infection	The ratio of MP infection (%)
With history of allergy (n = 51)	17	33.3
Without history of allergy (n = 125)	21	16.8
Total	38	21.6

χ2 = 5.849, p<0.05.

Of the 29 patients with bacterial infection, 10 (5.6%) patients had streptococcus pneumoniae, five (2.8%) patients had klebsiella pneumoniae, five (2.8%) patients had staphylococcus, three (1.7%) patients had E. coli, three (1.7%) patients had staphylococcus aureus, one (0.6%) had acid-producing bacillus, one (0.6%) had haemophilus influenzae, and one (0.6%) had acinetobacter baumannii. Seven (4.0%) patients were infected with a fungus, including six (3.4%) children with candida albicans and one with candida glabrata (**Figure 2**).

### Chest X-Ray Results and Wheezing

Chest X-rays at the time of evaluation were normal in five of 176 subjects from Xiangya Hospital. Forty subjects showed increased bronchovesicular shadows, of which one case was diagnosed with combined emphysema. One hundred twenty-two patients showed streaky shadows in X-ray examination results: two individuals combined with axillary lymph node, one subject combined with plural effusion, one had plural reaction, one had emphysematous left lung, and one had left-sided diaphragmatic hernia.

### Comparison of lgE, IL-4, IFN-γ, IL-4/IFN-γ, MMP3, MMP9, IL-17A, and IL-17E Levels in Different Groups of Cases From Xiangya Hospital and Hunan Children’s Hospital

The children with wheezing diseases had a slightly elevated serum IgE level compared with the control group [18.80(13.65-31.00) vs. 17.9(10.15-21.60), *p*=0.029]. There was no difference between the first wheezing group and the recurrent wheezing group [19.05(14.28-36.78) vs. 21.30(15.40-49.03), *p*>0.05]. Wheezing diseases were associated with significantly higher levels of IL-4 [24.00(24.00-48.00) vs. 23.00(9.50-27.00), *p*=0.0001] and IFN-γ [70.59(41.63-116.46) vs. 49.83(29.58-81.74), *p*=0.004] than the normal control. But there was no statistical difference in IL-4/IFN-γ (1.24 ± 1.88 vs. 0.68 ± 0.74, *p*>0.05) in the above groups. We also analyzed the difference between the first cases and recurrent wheezing cases, but they showed no difference in the level of IL-4 and IFN-γ and the ratio of IL-4/IFN-γ (*p*>0.05). Moreover, infants with wheezing diseases had significantly higher levels of MMP3 [53.40(20.02-128.2) vs. 30.90(13.80-50.95)] and MMP9 [148.10(99.30-276.10) vs. 122.10(82.20-162.35)] than the no-wheezing control (*p*=0.001). The subjects with recurrent wheezing showed higher levels of MMP9 than the samples with first wheezing [254.30(188.00-577.95) vs. 145.55(93.70-279.08), p=0.009], but there were no differences in the levels of MMP3. The difference of IL-17A [80.55(54.46-113.08) vs. 61.11(29.43-93.87)] and IL-17E [1.75(0.66-2.77) vs. 1.19(0.488-2.1615)] were only found in the comparison between wheezing groups and the control group *(p*=0.005, *p*=0.031) (**Table 5**).

**Table 5:** IgE, Interleukin- 4 (IL-4), interferon -γ (IFN-γ), IL-4/IFN-γ, MMP3, MMP-9, IL-17A, and IL-17E expression in 52 wheezing cases and 25 controls.

	No-wheezing control (n=25)	Wheezing children				*p*-values
		Total (n = 52)	First wheezing (n = 26)	Recurrent wheezing (n = 26)	*p*-values	
lgE [M (P_25_-P_75_) IU/mL]	17.9 (10.15-21.60)	18.80 (13.65-31.00)	19.05 (14.28-36.78)	21.30 (15.40-49.03)	0.627^a^	0.029^b^
IL-4 [M (P_25_-P_75_) pg/mL]	23.00 (9.50-27.00)	24.00 (24.00-48.00)	24.00 (24.00-155.00)	24.00 (24.00-109.50)	0.623^a^	0.0001^b^
IFN-γ [M (P_25_-P_75_) pg/mL]	49.83 (29.58-81.74)	70.59 (41.63-116.46)	92.39 (59.71-125.43)	90.68 (36.42-131.60)	0.770 ^a^	0.004^b^
IL-4/IFN-γ (x ± s)	0.68 ± 0.74	1.24 ± 1.88	0.98 ± 1.00	1.49 ± 2.47	0.194^c^	0.160^d^
MMP3 [M (P_25_-P_75_) pg/mL]	30.90 (13.80-50.95)	53.40 (20.02-128.2)	65.72 (24.85-137.75)	94.15 (38.81-213.23)	0.564^a^	0.001^b^
MMP9 [M (P_25_-P_75_) pg/mL]	122.10 (82.20-162.35)	148.10 (99.30-276.10)	145.55 (93.70-279.08)	254.30 (188.00-577.95)	0.009^a^	0.001^b^
IL-17A [M (P_25_-P_75_) pg/mL]	61.11 (29.43-93.87)	80.55 (54.46-113.08)	96.07 (66.66-130.13)	83.11 (54.60-135.46)	0.301^a^	0.005^b^
IL-17E [M (P_25_-P_75_) pg/mL]	1.19 (0.488-2.1615)	1.75 (0.66-2.77)	1.83 (1.04-3.47)	2.13 (0.48-3.96)	0.687^a^	0.031^b^

**^a^** Rank sum test for comparison of first wheezing group versus recurrent wheezing group.

**^b^** Rank sum test for comparison of wheezing group versus no-wheezing control.

**^c^** ANOVA for comparison between first wheezing and recurrent wheezing patients.

**^d^** ANOVA for comparison between no-wheezing control and wheezing infants.

In recent years, the incidence of wheezing is increasing year by year. Over half of children wheezing break out repeatedly, and breathing and the immune system to mature in infants and young children period, the period of recurrent wheezing may help children to adversely affect the body’s immune system and respiratory system, after treatment the most infant wheezing can alleviate, but about 50% of children with recurrent wheezing can develop bronchial asthma for children. As the symptoms of wheezing and the corresponding risk factors in children change over time, there are certain limitations. Therefore, it is important to explore the association between the pathogenesis of wheezing diseases and the pathogenesis of asthma. The essence of pediatric bronchial asthma is chronic airway inflammation, and Th1/Th2 imbalance is a widely accepted theory. MMP3, MMP9, and IL-17 are involved in airway remodeling of childhood asthma, and in our study they are also involved in the pathogenesis of infant wheezing disease, which provide more experimental basis for prevention and treatment of infant wheezing diseases.

## Discussion

Wheezing disease was one of the most common respiratory diseases with greater prevalence in winter and spring. Patients can develop heart failure and the illnesses are life threatening if they were not treated properly. Most of the wheezing children were under 3 years old and boys were predominant in the proportion of patients. Previous studies showed that wheezing infants usually had a positive history of allergy (eczema, allergic rhinitis, urticaria, and food allergies) individually or in their family, and a similar result was found in our research (13).

It was of note that children with eczema and papule-like urticaria were more likely to develop asthma. Zhao et al. had noted allergic history and asthma history in the family as risk factors for treating asthma (14). Ronmark et al. carried out a survey with a questionnaire with 30,000 subjects in Swiss suggesting that the co-existence of asthma, rhinitis, and eczema is common. Allergy, asthma history in the family, and smoking were all risk factors for eczema and the patients were characterized by wheezing. Based on our own result and Ronmark’s research, allergy history was closely associated with wheezing diseases and was a risk factor for wheezing episodes (15). Fifty-one of 176 children in our research had allergy history such as eczema, allergic rhinitis, urticaria, milk allergy, and drug allergy. Of the first wheezing patients, 21.3% had an allergy history and the rate was significantly higher in the subgroup of recurrent wheezing infants. Therefore, it is conceivable that allergy history was an independent risk factor for wheezing disease.

In our cohort, over half of the patients had an airway infection, including 29.6% of the patients tested had a viral infection, 21.6% of them had MP infection, and 32 subjects had more than one infection. Dominant amounts of major pathogens were restricted to mycoplasma. In our study, 21.6% of the patients who were hospitalized for asthma had MP infection, which was a basic coincidence with the previous report (16). We also conducted a study to determine the association between MP infection and allergy history. Our results showed a different proportion of MP infection between subjects with and without allergy history.

In our study, 29.6% of all the subjects were infected with a virus, 27 patients had coxsackie virus (15.3% of all the samples), 13 patients had RSV infection (6.8% of all the samples), and 12 children had cytomegalovirus (6.8% of all the samples). The discrepancy observed between our result and the previous study may result from the different times of sample collection, as our samples were collected in winter.

Viral infection at an early age is linked to recurrent wheezing diseases and asthma in children. Viral infection causes the destruction of airway epithelial cells and results in the decrease of the airway immune response, and indirectly exacerbates allergic inflammation. It is suggested there were more eosinophils and lymphocytes infiltration in the lung tissues within RSV-infected mice. Besides, the increased level of cytokine TNF-α, IFN-γ, IL-5, and IL-2 were also observed (17). RSV infection had an influence on Th1/Th2 compartment toward Th1 and facilitated the development of airway inflammation. Some studies have reported that RSV-infected airway epithelial cells secrete cytokines and chemokines including thymic stromal lymphopoietin which work to activate Th2 and lead to Th2 factor secretion. RSV infection also activates T lymphocyte like Th2, Th17, regulatory T cells, and cytotoxic T cells (18). The results of Tourdot’s research showed that RSV infection in mice would not induce airway inflammation but would thicken the mouse bronchial basement membrane and result in an increased amount of collagen in lung tissue. In addition, the amount of fibroblastic growth factor (FGF) was increased with RSV infection (19).

Previous studies have identified the association between wheezing diseases and viral infection, but the role of bacterial infection has not been shown. One study determined Haemophilus influenzae, Moraxella catarrhalis, and Streptococcus pneumoniae as the major causes of bacterial infection associated with wheezing. In this research, gram-negative bacilli were found in most of the samples, but the number of white blood cells and neutrophils was sometimes not increased. The bacterial airway colonization was reported to increase the risk of wheezing and continuous wheezing leading to the exacerbation of acute wheezing diseases (20). Of all the samples in our present study, 16.5% were infected with bacterial-like streptococcus pneumoniae, klebsiella pneumoniae, staphylococcus, Escherichia coli, staphylococcus aureus, acid-producing bacillus, haemophilus influenzae, and acinetobacter baumannii, indicating that bacterial infection was associated with wheezing diseases of infants.

A lower respiratory tract viral infection with an upper respiratory tract bacterial infection was prevalent among wheezing patients. In the study cohort of Hishiki’s research (21), 43.6% of all the patients had bacterial infections, haemophilus influenzae (43.9% of all the samples), streptococcus pneumoniae (36.6% of all the samples), and moraxella catarrh (29.3% of all the samples) were the common causative bacteria. RSV-infected wheezing children were often co-infected with a drug-resistant lower respiratory tract infection (21). A previous study, which enrolled 165 RSV-infected children who lived in an intensive care unit, suggested that gram-positive bacteria was found in the lower respiratory tract secretion in 42.4% of all the samples. The RSV-infected children tended to develop bacterial pneumonia (22). Besides virus and bacterial coinfection, the coinfection of different kinds of viruses was also observed in patients.

The wheezing patients were often during mixed infection; however, the mechanism causing wheezing was not thoroughly studied. One possible explanation is that patients with MP or virus may suffer airway mucosal cell damage, thus making them more likely to be infected with another pathogen. 

In our study, 32 subjects were infected with more than two kinds of pathogens (18.2% of all the patients), six individuals were infected with three pathogens (3.4% of all the samples), and 18.8% of the patients had mixed infection means “had more than one infection. Virus and MP infected all six patients. It reminded us of the importance of combination therapy for these patients.

There were reports that showed that the mechanism of immune response in children with allergic rhinitis was similar to that of asthma. Recurrent eczema was a common allergic disease characterized by Th1/Th2 imbalance. Increased levels of lgE was detected in patients with eczema and the level of lgE was positively correlated with the severity of the disease. In our cohort from Hunan Children’s Hospital, the level of lgE was significantly higher in the wheezing group but was not associated with the number of wheezing episodes.

IL-4 was specifically secreted by Th2 cells and worked to promote the proliferation, differentiation, and activation of B cells. It was critical in the synthesis of lgE by B cells and was associated with immune diseases. Another important cytokine in Th1/Th2 balance was IFN-γ, which was a cytokine signature of activated T cells and NK cells. IFN-γ functioned to activate macrophages, neutrophils, and NK cells, as well as to suppress the proliferation of Th2 cells by promoting the transition of Th0 cells to Th1 cells. Some experts thought IFN-γ and IL-12 could prevent damage from RSV infection (23), but there were studies showing that the level of IFN-γ varied with age because increased IFN-γ expression was observed in patients who were older than 1 year old compared to a healthy control , comparing with age-matched controls, IFN-γ levels were significantly higher in RSV group ≥12 months of age (1 year old). Our result showed that elevated IFN-γ levels may be age-related (average age in the wheezing group was 12.65 ± 8.68 months) (24). Our results showed that the level of IL-4 and IL-4/IFN-γ was significantly increased in wheezing children, indicating the activation of Th1 and Th2 cells. However, in our study, the level of IFN-γ was increased in wheezing children. To our knowledge, the discrepancy may relate to age.

Biopsy examination for severe asthma airway tissue showed the increased level of MMP9 (25). MMP9 could stimulate the secretion of growth factor like TGF-β1 and FGF and induce cell proliferation and differentiation. MMP9 was also an essential player in IL-13 mediating TGF-β1 secretion and was involved in pulmonary fibrosis and degradation of extracellular matrix. MMP served as a marker of airway remodeling (26). A previous study suggests that RSV infection may be associated with bronchial hyper-responsiveness, as leukotriene, neutrophil, and lymphocyte levels increased in alveolar lavage fluid of infected mice and bronchial hyper-responsiveness is observed in mice (27). MMP3 cannot only synergistically degrade the extracellular matrix, but also accelerate the decomposition of the basement membrane of pulmonary blood vessels, and participate in the remodeling of pulmonary blood vessels, playing an indispensable role in the occurrence and prognosis of lung diseases. In mouse models, it was found that MMP3 can also mediate the occurrence of acute lung injury by interacting with neutrophils and macrophages (28). We evaluated the expression of MMP3 and MMP9 and found a significant increase in wheezing patients. Moreover, the level of MMP9 was associated with the number of wheezing episodes as there was a difference in our study between first and recurrent wheezing children.

IL-17 was another altered cytokine in wheezing children compared to the control group. In our analysis, both IL-17A and IL-17E were significantly increased in the wheezing group, but there was no difference between first and recurrent wheezing patients. IL-17 was a major pro-inflammation cytokine produced by Th17 cells. IL-17 had been recognized to induce the production of Th2-specific cytokine-like IL-4 and IL-13 and was also involved in Th1/Th2 imbalance (29). Our study revealed that IL-17 is involved in the pathogenesis of wheezing disease in young children, and children with a wheezing disease may experience airway inflammation and airway remodeling.

It should be noted that limitations exist in this study. Firstly, the sample size was relatively small to analyze the association between cytokines and wheezing disease, which may lead to low statistical power for data analysis. Thus it is necessary to strengthen cooperation between hospitals. Secondly, our study was preliminary, and more research is needed to explore the mechanisms of wheezing disease, especially in immune response.

## Conclusions

In our study, a higher incidence of wheezing disease was observed in boys than girls and wheezing disease was prevalent in children under 3 years old. Children with allergy history were more likely to develop wheezing diseases. MP and viral infection were the common causative pathogens of infants with wheezing diseases and infants with allergy history were more vulnerable to MP infection. MMP3, MMP9, and IL-17 are involved in the pathogenesis of children wheezing disease, which provide a more experimental basis for the prevention and treatment of wheezing disease.

## Data Availability Statement

The original contributions presented in the study are included in the article/supplementary material. Further inquiries can be directed to the corresponding author.

## Ethics Statement

The studies involving human participants were reviewed and approved by Chinese Clinical Trial Registry. Written informed consent to participate in this study was provided by the participants’ legal guardian/next of kin.

## Author Contributions

YJT and RHH prepared original draft preparation, YJT and YXY analyzed data, XRZand CTL collected sample, RH supervised the whole study, reviewing and editing the final approval of the version submitted for publication.

## Funding

This work was supported by the Hunan Provincial Natural Science Foundation of China (2016JJ4109 and 2020JJ4909).

## Conflict of Interest

The authors declare that the research was conducted in the absence of any commercial or financial relationships that could be construed as a potential conflict of interest.

## Publisher’s Note

All claims expressed in this article are solely those of the authors and do not necessarily represent those of their affiliated organizations, or those of the publisher, the editors and the reviewers. Any product that may be evaluated in this article, or claim that may be made by its manufacturer, is not guaranteed or endorsed by the publisher.
